# Apparent Diffusion Coefficient and Native T1 Mapping Histogram Analyses Reveal Tumor Proliferation and Microenvironment in Neuroblastoma Xenografts

**DOI:** 10.3390/cancers17213433

**Published:** 2025-10-26

**Authors:** Haoru Wang, Xiang Cheng, Qian Hu, Lisha Nie, Weiyi Zhu, Yingxue Tong, Xin Chen, Ling He, Huiru Zhu, Jie Huang, Jiaxin Su, Chen Zeng, Jinhua Cai

**Affiliations:** 1 Department of Radiology, Children’s Hospital of Chongqing Medical University, National Clinical Research Center for Child Health and Disorders, Ministry of Education Key Laboratory of Child Development and Disorders, Chongqing Key Laboratory of Child Neurodevelopment and Cognitive Disorders, Chongqing 400014, China; 2 GE Healthcare, MR Research, Beijing 100076, China; 3 National Clinical Research Center for Child Health and Disorders, Children’s Hospital of Chongqing Medical University, Chongqing 400014, China

**Keywords:** neuroblastoma, native T1 mapping, tumor microenvironment, cell proliferation, apparent diffusion coefficient

## Abstract

**Simple Summary:**

Neuroblastoma is a common childhood cancer, and understanding how it grows and interacts with its surrounding environment is important for developing better treatments. Magnetic resonance imaging (MRI) can provide detailed information about tumors without surgery, but it is still unclear which MRI measurements best reflect tumor activity and structure. In high-risk neuroblastoma, upfront surgery is rarely performed, and neoadjuvant chemotherapy is often required, making direct correlation between imaging and biopsy challenging. In this exploratory preclinical study, we used advanced MRI techniques, including apparent diffusion coefficient (ADC) and native T1 mapping, in a mouse model of neuroblastoma to explore how these imaging measures relate to tumor proliferation and microenvironment. We found that ADC values were negatively related to tumor cell growth, whereas T1 mapping features were positively related to collagen content after false discovery rate correction. These findings provide preliminary evidence that combining ADC and T1 mapping can capture complementary aspects of neuroblastoma biology. The complementary information derived from ADC and T1 mapping may contribute to noninvasive assessment of tumor aggressiveness, extracellular remodeling, and risk stratification, providing a methodological foundation for future diagnostic and prognostic applications in clinical neuroblastoma imaging.

**Abstract:**

**Objectives**: This exploratory preclinical study aimed to compare the correlations of apparent diffusion coefficient (ADC) and native T1 mapping histogram features with tumor cell proliferation, microvessel density (MVD), and extracellular matrix composition in neuroblastoma xenografts. **Methods**: Neuroblastoma xenografts (*n* = 42) were established by subcutaneously injecting three *MYCN*-amplified/non-amplified human neuroblastoma cell lines (IMR-32, SK-N-BE(2), and SH-SY5Y; *n* = 14 per group) into female immunodeficient BALB/c-nude mice. Once tumors reached a diameter within the range of 12–15 mm, native T1 mapping and diffusion-weighted imaging were performed using a 3.0T clinical MRI scanner. Tumor cell proliferation and MVD were assessed via immunohistochemical Ki-67 staining and CD31 staining, respectively. Collagen fibers were visualized using Masson staining to calculate the collagen volume fraction (CVF). Pearson correlation coefficients with false discovery rate (FDR) correction were used to evaluate their associations. **Results**: Significant negative correlations were observed between Ki-67 expression and multiple ADC values after FDR correction, including ADC_10Percentile_ (*r* = −0.397, adjusted *p* = 0.032), ADC_90Percentile_ (*r* = −0.394, adjusted *p* = 0.032), ADC_maximum_ (*r* = −0.362, adjusted *p* = 0.048), ADC_mean_ (*r* = −0.421, adjusted *p* = 0.032), ADC_median_ (*r* = −0.422, adjusted *p* = 0.032), ADC_minimum_ (*r* = −0.390, adjusted *p* = 0.032), and ADC_rootmeansquared_ (*r* = −0.419, adjusted *p* = 0.032). In contrast, multiple T1 mapping features showed significant positive correlations with CVF (adjusted *p* < 0.05). **Conclusions**: ADC and T1 mapping provide complementary insights into tumor proliferation and extracellular matrix composition in neuroblastoma. These preclinical findings support further research to validate their potential clinical utility.

## 1. Introduction

Neuroblastoma is a clinically heterogeneous pediatric cancer, with high-risk cases demonstrating poor long-term survival despite intensive multimodal therapy [1,2]. Tumor cell proliferation, often driven by *MYCN* amplification and dysregulated cell-cycle control, represents a major determinant of tumor aggressiveness and clinical outcome [3]. Although current risk stratification frameworks integrate clinical, genetic, and histopathological factors, they remain suboptimal in accurately predicting prognosis and guiding personalized therapy [4]. This highlights the need to identify novel biomarkers that can refine risk classification in neuroblastoma. Increasing evidence highlights the importance of the tumor microenvironment (TME), including the vasculature and extracellular matrix (ECM), in driving neuroblastoma progression [5,6]. Microvessel density (MVD) correlates with tumor viability and differentiation [7], while ECM composition influences invasive behavior and metastatic potential [8,9]. Specifically, tumors with poor prognosis often exhibit denser ECM organization, whereas favorable-outcome tumors display looser ECM structures [10]. Moreover, ECM profiles may identify ultra-high-risk subgroups within the high-risk population [11]. Despite its clinical relevance, TME characterization currently relies largely on invasive biopsy, which constrains its applicability for noninvasive and longitudinal monitoring, especially in patients undergoing neoadjuvant chemotherapy. Consequently, the development of noninvasive imaging biomarkers reflecting TME characteristics holds considerable promise for elucidating TME heterogeneity in neuroblastoma and facilitating the evaluation of emerging therapeutic strategies [12].

Magnetic resonance imaging (MRI), which does not involve ionizing radiation, is well suited for pediatric oncologic imaging. In addition to anatomical evaluation, MRI offers functional parameters [13]; for instance, the apparent diffusion coefficient (ADC), derived from diffusion-weighted imaging (DWI), reflects tumor cellularity and histological characteristics [14,15]. However, the extent to which ADC is influenced by TME components, including MVD and ECM, remains poorly understood, limiting its utility as a surrogate biomarker for TME attributes in neuroblastoma. Conventional imaging also demonstrates limited utility in assessing the clinical efficacy of anti-GD2 immunotherapy in high-risk neuroblastoma, highlighting the need for more advanced MRI approaches [16]. Native T1 mapping, a non-contrast MRI technique that quantifies tissue T1 relaxation times, has been associated with ECM-related features including fibrosis and collagen content [17,18]. Despite this potential, its application in pediatric tumors is limited, with most evidence derived from adult cancers [19,20,21]. A recent preclinical study using 7.0T MRI showed associations between T1 values and treatment response in neuroblastoma xenografts [22], yet clinical validation remains absent. Furthermore, conventional mean-value analysis may obscure intratumoral spatial heterogeneity. Histogram-based analysis of ADC and T1 mapping enables voxel-wise assessment and extraction of metrics such as skewness, kurtosis, and entropy, offering a more detailed characterization of tumor heterogeneity [23]. A previous study has shown that ADC histogram features can distinguish between *MYCN*-amplified and non-amplified neuroblastomas, though the underlying biological mechanisms remain unclear [24]. These advanced analytical approaches offer a promising framework for noninvasive assessment of both tumor cellularity and TME characteristics, with potential applications in treatment monitoring and risk stratification.

The relatively low incidence of neuroblastoma (11–13 cases per million children annually) [25] and the limited frequency of *MYCN* amplification (~20% of cases) [26] hinder large-scale clinical radiologic–pathologic studies. Moreover, in high-risk cases, where upfront surgery is rarely the first option and neoadjuvant chemotherapy is typically required, accurately correlating MRI findings with biopsy specimens is technically challenging, limiting radiology–pathology studies in patients. To address this gap, we conducted an exploratory preclinical study in mouse xenograft models, aiming to correlate histogram-based ADC and native T1 mapping features with tumor cell proliferation and microenvironment features in neuroblastoma. Additionally, we used *MYCN*-amplified and non-amplified xenograft models to evaluate how genetic background influences imaging phenotypes and TME composition.

## 2. Materials and Methods

### 2.1. Ethics Approval

The animal use protocol for this study was reviewed and approved by the Ethics Committee of Children’s Hospital of Chongqing Medical University (Approval Number: CHCMU-IACUC20241101002).

### 2.2. Selection and Culturing of Cell Lines

Three representative human neuroblastoma cell lines were used in this study: SH-SY5Y, IMR-32, and SK-N-BE(2). SH-SY5Y is a *MYCN*-non-amplified cell line, whereas IMR-32 and SK-N-BE(2) are *MYCN*-amplified [27,28,29]. All cell lines were obtained from Wuhan Procell Life Science & Technology Co., Ltd. (Wuhan, China) and authenticated by short tandem repeat profiling. Cells were cultured in base medium supplemented with 10% fetal bovine serum and 1% penicillin/streptomycin at 37 °C in a humidified incubator with 5% CO_2_. Cells were passaged at 80–90% confluence using 0.25% trypsin–EDTA (Gibco, Thermo Fisher Scientific, Grand Island, NY, USA).

### 2.3. Calculation of Sample Size for Experimental Animals

The minimum required sample size for correlation analysis was estimated using a standard formula based on the expected Pearson correlation coefficient: $n={\left(\frac{Z_{\alpha /2}+Z_{\beta }}{r}\right)}^{2}$. Referencing prior research that reported a correlation coefficient of approximately 0.64 between T1 values and extracellular matrix content in a rabbit hepatic cancer model [17], we set the expected *r*-value to 0.50. This yielded a minimum requirement of 32 mice to achieve 80% power at a 0.05 significance level. To account for potential data loss, the sample size was increased by 10–20%. Given the use of three human neuroblastoma cell lines, 14 mice were allocated per group, resulting in a total of 42 animals.

### 2.4. Establishment of Xenograft Models

Forty-two female immunodeficient BALB/c-nude mice (4–6 weeks old; body weight 16–20 g) were purchased from Chongqing Enbi Biotechnology Co., Ltd. (Chongqing, China). All animals were housed in a specific pathogen-free environment under controlled conditions (temperature: 22 ± 2 °C; humidity: 50 ± 10%; 12-h light/dark cycle), with free access to sterilized food and water. After a one-week acclimatization period, the mice were used to establish xenograft models. The mice were randomly allocated into three groups (*n* = 14 per group), with each group corresponding to one of the human neuroblastoma cell lines (IMR-32, SK-N-BE(2), or SH-SY5Y). Randomization was performed using a computer-generated random number sequence to minimize selection bias.

For tumor implantation, a suspension of 3 × 10^6^ cells in 100 µL phosphate-buffered saline was subcutaneously injected into the right flank of each mouse using a sterile 1-mL syringe. Mice were monitored daily for general health, behavior, and tumor development. Tumor dimensions were measured every 2–3 days using a caliper, and tumor volume was calculated using the formula: (length × width^2^)/2. In our preliminary experiments, tumors smaller than 10 mm frequently produced distorted DWI images, resulting in poor image quality on both DWI and ADC maps. In accordance with animal welfare regulations, the maximum permissible tumor diameter was set at 15 mm. Therefore, imaging was performed once tumors reached a diameter within the range of 12–15 mm. After MRI scanning, tumors were surgically excised and processed for histopathological analysis. Dropouts were documented and excluded from analysis if mice failed to develop measurable tumors, if tumors ulcerated, or if animals died from unrelated causes before imaging. The final number of animals included in the analysis was forty-two.

### 2.5. MRI Examination

MRI examinations were performed using a 3.0T clinical scanner (Discovery MR750, GE Healthcare, Waukesha, WI, USA) equipped with an 8-channel rat coil. Mice were anesthetized with an intraperitoneal injection of Delivector™ Avertin (20 µL/g, Dowobio, Shanghai, China). The protocol included axial T1-weighted imaging (T1WI), T2-weighted imaging (T2WI), DWI, and native T1 mapping. The system was equipped with GE Healthcare’s IQ Engine software to accelerate acquisition while preserving image quality.

For T1WI, the repetition time (TR) was 500.0 ms, echo time (TE) was the minimum full, field of view (FOV) was 80 × 80 mm^2^, slice thickness was 2 mm, slice number was 10, with no spacing between slices, matrix size was 192 × 192, and the voxel size was 0.4 × 0.4 × 2.0. T2WI parameters included a TR of 2600.0 ms, TE of 102.0 ms, FOV of 80 × 80 mm^2^, slice thickness of 2 mm, slice number of 10, no slice spacing, matrix size of 256 × 224, and a voxel size of 0.3 × 0.4 × 2.0. For PROPELLER DWI, TR was set at 3500.0 ms, TE at 62.0 ms, FOV at 100 × 100 mm^2^, slice thickness at 3 mm, slice number at 8, no slice spacing, matrix size of 128 × 128, and voxel size of 0.8 × 0.8 × 3.0. DWI was acquired with b-values of 0 and 800 s/mm^2^ to balance diffusion sensitivity with image quality, as higher b-values reduce signal-to-noise ratio and increase susceptibility to motion artifacts.

Native T1 mapping was performed using a 3D variable flip angle (VFA) spoiled gradient-recalled echo (SPGR) sequence with flip angles of 6°, 9°, 12°, and 15°, enabling rapid imaging while minimizing respiratory motion artifacts. Key parameters included TR = 4.4 ms, TE = 2.2 ms, FOV = 100 × 100 mm^2^, slice thickness = 2 mm, 10 slices, matrix size = 160 × 160, and voxel size = 0.6 × 0.6 × 2.0. By acquiring multiple gradient-echo images at different flip angles, the VFA approach allowed generation of native T1 relaxation maps [18,30].

The approximate acquisition times were as follows: T1WI (1 min 28 s), T2WI (1 min 26 s), DWI (2 min 31 s), and native T1 mapping (64 s).

### 2.6. Mice Sacrifice and Histopathological Analysis

Following MRI scanning, mice were euthanized by cervical dislocation within one hour, and the subcutaneous tumors were surgically excised. Each tumor was sectioned along the plane corresponding to its largest cross-sectional area on MRI. Analyses were focused on the central largest tumor slice, with ADC and native T1 values measured on this section. Corresponding histological staining was performed on the same slice. Tissue samples were immediately fixed in 4% paraformaldehyde for 24–48 h to preserve structural integrity. After fixation, the samples were dehydrated through a graded ethanol series, cleared in xylene, and embedded in paraffin. Serial sections of 4 µm thickness were cut using a microtome, mounted on glass slides, and subjected to histopathological analysis. For each tumor, three consecutive sections were stained, including Ki-67, CD31, and Masson’s trichrome, all derived from the central plane.

Ki-67, CD31, and Masson staining are widely used histological techniques for evaluating tumor cell proliferation, MVD, and collagen fibers, respectively. For quantitative analysis of Ki-67 and Masson staining, five randomly selected fields of view were captured from each slide at 400× magnification for Ki-67 and 200× for Masson staining. For quantitative analysis of CD31 staining, the three most vascularized “hotspot” areas were first identified under low magnification (100×), and then the number of brown-stained vascular endothelial cells was counted under higher magnification (200×) [31]. Fiji ImageJ software (version 1.53q; https://imagej.net/ij, accessed on 22 October 2024, National Institutes of Health, Bethesda, MD, USA) was used to analyze the stained sections. The Ki-67 index was calculated as the percentage of Ki-67 positive cells relative to the total number of cells in each field. Similarly, the collagen volume fraction (CVF) was quantified by measuring the area of blue-stained collagen fibers in the Masson-stained sections. MVD was calculated as the average number of brown-stained microvessels counted across the three hotspot regions.

### 2.7. ADC and Native T1 Map Histogram Analysis

ADC and native T1 maps were generated using the ADW 4.6 post-processing workstation (GE Healthcare, USA). Voxel-wise T1 maps were computed by fitting the SPGR steady-state signal equation to multi-flip-angle images (6°, 9°, 12°, 15°):

$$S(\alpha ,TR)=M_{0}exp(-TE/T_{2}^{*})\frac{\left(1-E_{1}\right)\sin\left(\alpha \right)}{1-E_{1}\cos\left(\alpha \right)},\;\mathrm{where}\;E_{1}=exp(-\frac{TR}{T_{1}})$$

All flips used identical short TE. No voxel-wise B1 mapping was performed (α_eff_ = α). Fitting used voxel-wise nonlinear least-squares Levenberg–Marquardt algorithm. When the original ADC and native T1 maps were reconstructed by the workstation, pseudocolor maps were generated by applying a colormap to the same quantitative data to improve visualization. The original ADC and native T1 maps were then imported into 3D Slicer (version 5.6.1) for manual region of interest (ROI) delineation. A pediatric radiologist with six years of experience in pediatric oncology manually outlined the tumor margins on the largest axial slice using the “Segment Editor” module.

**Figure 1:**
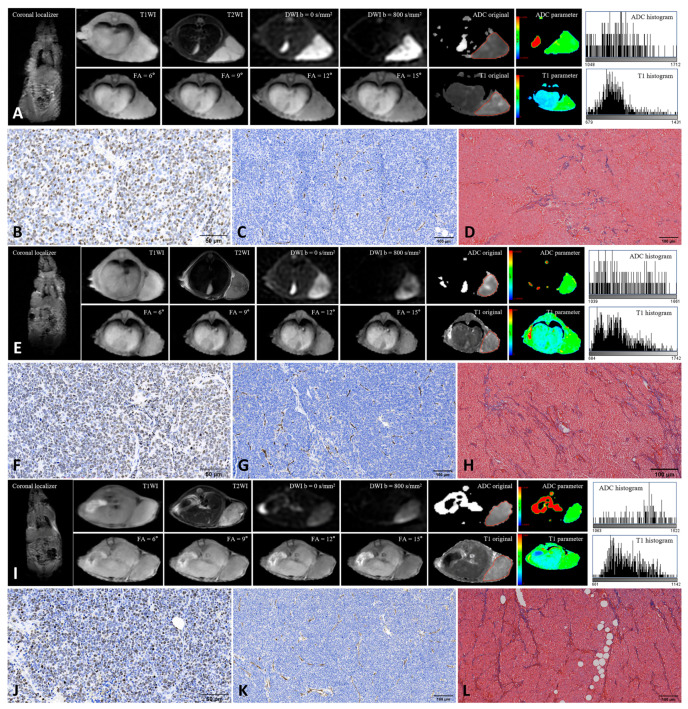
Representative MRI and histopathological images of three neuroblastoma xenografts. (**A**–**D**): SH-SY5Y; (**E**–**H**): IMR-32; (**I**–**L**): SK-N-BE(2). Panels show MRI images (**A**,**E**,**I**), Ki-67 staining (**B**,**F**,**J**), CD31 staining (**C**,**G**,**K**), and Masson’s trichrome staining (**D**,**H**,**L**). Red lines along the tumor margin indicate the manually drawn region of interest used for histogram analysis. Ki-67 staining was performed at 400× magnification, and CD31 and Masson’s trichrome staining at 200× magnification. Positive staining is indicated by brown nuclei for Ki-67, brown microvessels for CD31, and blue collagen fibers for Masson staining.

From each ROI, 18 first-order histogram features (10Percentile, 90Percentile, Energy, Entropy, InterquartileRange, Kurtosis, Maximum, MeanAbsoluteDeviation, Mean, Median, Minimum, Range, RobustMeanAbsoluteDeviation, RootMeanSquared, Skewness, TotalEnergy, Uniformity, Variance) were extracted from both original ADC and native T1 maps using the PyRadiomics package (version 3.1.0) (Table 1). To assess interobserver reproducibility, a second pediatric radiologist with more than ten years of experience independently repeated the ROI delineation and feature extraction following the same standardized protocol. Interobserver agreement was evaluated to ensure measurement reliability. Figure 1 shows the MRI images and TME features of the xenografts derived from the three human neuroblastoma cell lines.

### 2.8. Statistical Analysis

Statistical analyses were conducted using RStudio (version 4.4.2). The normality of quantitative data was assessed with the Shapiro–Wilk test, and data are presented as mean ± standard deviation (SD). For intergroup comparisons, one-way analysis of variance (ANOVA) was applied when the assumption of homogeneity of variance was met, while the Kruskal–Wallis test was used for data with unequal variances. Post hoc analyses were conducted for variables showing significant intergroup differences: Tukey’s Honestly Significant Difference test was applied under homogenous variance conditions, whereas Dunn’s test with Benjamini–Hochberg adjustment was used when variances were heterogeneous.

Pearson correlation analysis with false discovery rate (FDR) correction was employed to evaluate the relationships between imaging-derived features, Ki-67 expression, MVD, and CVF. Interobserver agreement for the extracted histogram features was assessed using intra-class correlation coefficients (ICCs), calculated via a two-way random-effects model. Bland–Altman plots were used to visualize the agreement between the two sets of measurements. ICC values were interpreted as follows: <0.50, poor; 0.50–0.75, moderate; 0.75–0.90, good; and >0.90, excellent reliability [32]. A two-tailed *p*-value less than 0.05 was considered statistically significant.

## 3. Results

### 3.1. Comparison of Ki-67 Expression, MVD, and CVF

The mean Ki-67 indices for the SH-SY5Y, IMR-32, and SK-N-BE(2) groups were 63.84% (SD: 10.56%), 76.83% (SD: 4.43%), and 72.66% (SD: 5.33%), respectively. Overall comparison among the three groups showed a significant difference (*p* = 0.002). Post hoc pairwise analysis revealed that the Ki-67 index was significantly higher in the IMR-32 group compared with the SH-SY5Y group (adjusted *p* = 0.001). However, post hoc pairwise comparison showed no significant difference between IMR-32 and SK-N-BE(2) (adjusted *p* = 0.087) or between SH-SY5Y and SK-N-BE(2) (adjusted *p* = 0.087)

The mean MVD values were 68 (SD: 17) for SH-SY5Y, 81 (SD: 13) for IMR-32, and 75 (SD: 16) for SK-N-BE(2). The overall comparison indicated significant differences among the three groups (*p* = 0.029). In pairwise analyses, MVD was significantly higher in the IMR-32 group compared with the SH-SY5Y group (adjusted *p* = 0.022). Post hoc pairwise comparison showed no significant differences between IMR-32 and SK-N-BE(2) (adjusted *p* = 0.276) or between SK-N-BE(2) and SH-SY5Y (adjusted *p* = 0.450).

For CVF, the mean values were 5.01% (SD: 0.99%) for SH-SY5Y, 5.08% (SD: 1.02%) for IMR-32, and 5.80% (SD: 0.58%) for SK-N-BE(2). The overall group comparison was statistically significant (*p* = 0.045). However, post hoc pairwise comparisons did not reveal statistically significant differences between any two groups (SH-SY5Y vs. IMR-32: adjusted *p* = 0.973; SK-N-BE(2) vs. IMR-32: adjusted *p* = 0.097; SK-N-BE(2) vs. SH-SY5Y: adjusted *p* = 0.061) (Supplementary Figure S1).

### 3.2. Comparison of ADC and Native T1 Mapping Histogram Features

**Figure 2:**
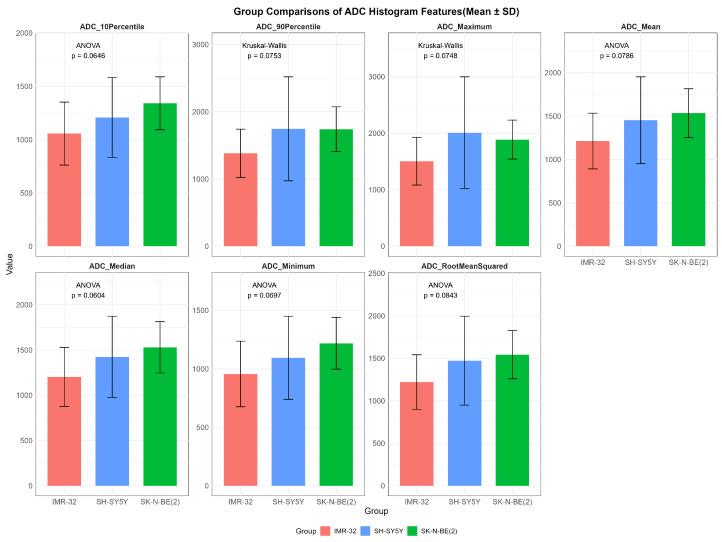
Comparisons of representative apparent diffusion coefficient histogram features between IMR-32, SH-SY5Y, and SK-N-BE(2) groups. The unit of apparent diffusion coefficient value is ×10^−6^ mm^2^/s.

While no statistically significant differences in ADC values were observed among the three groups (*p* > 0.05), several features showed overall potential trends toward variation, including ADC_maximum_ (*p* = 0.075), ADC_mean_ (*p* = 0.079), ADC_median_ (*p* = 0.060), ADC_minimum_ (*p* = 0.070), ADC_rootmeansquared_ (*p* = 0.084), ADC_10Percentile_ (*p* = 0.065), and ADC_90Percentile_ (*p* = 0.075) (Figure 2).

**Figure 3:**
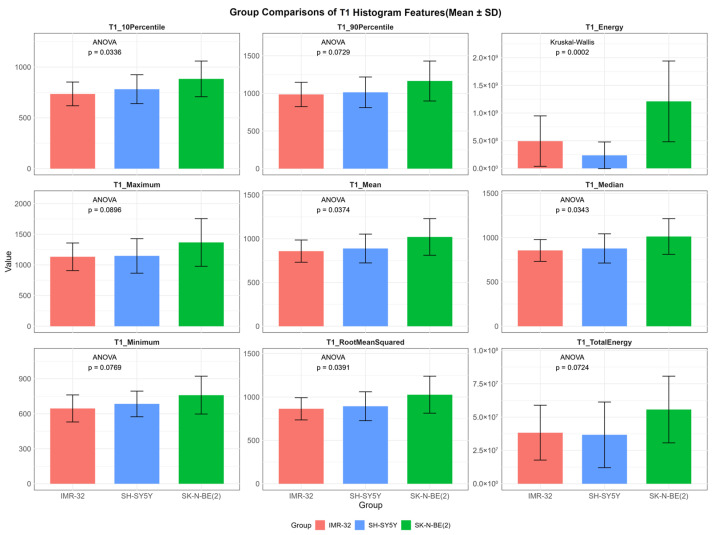
Comparisons of representative native T1 histogram features between IMR-32, SH-SY5Y, and SK-N-BE(2) groups. The unit of T1_energy_ and T1_totalenergy_ is ms^2^ and ms^2^ × mm^3^, respectively, and the unit of remaining features is ms.

In contrast, several T1 histogram features exhibited overall statistically significant differences among the three groups, including T1_energy_ (*p* < 0.001), T1_mean_ (*p* = 0.037), T1_median_ (*p* = 0.034), T1_rootmeansquared_ (*p* = 0.039), and T1_10Percentile_ (*p* = 0.034). Additionally, T1_maximum_ (*p* = 0.090), T1_minimum_ (*p* = 0.077), T1_totalenergy_ (*p* = 0.072), and T1_90Percentile_ (*p* = 0.073) demonstrated suggestive trends of variation (Figure 3). Post hoc pairwise analysis revealed significant differences between IMR-32 and SK-N-BE(2) in T1_10Percentile_ (adjusted *p* = 0.030), T1_energy_ (adjusted *p* = 0.008), T1_mean_ (adjusted *p* = 0.042), T1_median_ (adjusted *p* = 0.042), and T1_rootmeansquared_ (adjusted *p* = 0.044). A significant difference in T1_energy_ was also found between SH-SY5Y and SK-N-BE(2) (adjusted *p* = 0.002).

### 3.3. Correlation Between ADC Values, Ki-67 Expression, MVD, and CVF

**Figure 4:**
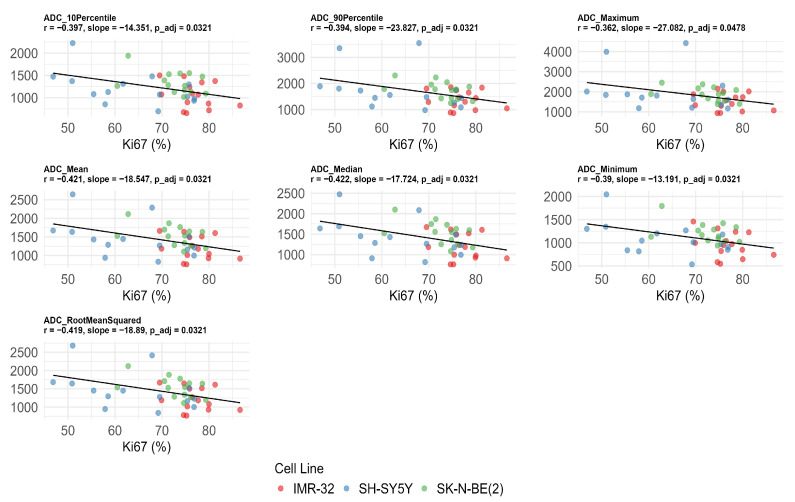
Significant negative correlations between apparent diffusion coefficient (ADC) histogram features and Ki-67 index after false discovery rate correction. The unit of ADC value is ×10^−6^ mm^2^/s.

Significant negative correlations were observed between Ki-67 expression and multiple ADC values after FDR correction, including ADC_10Percentile_ (*r* = −0.397, adjusted *p* = 0.032), ADC_90Percentile_ (*r* = −0.394, adjusted *p* = 0.032), ADC_maximum_ (*r* = −0.362, adjusted *p* = 0.048), ADC_mean_ (*r* = −0.421, adjusted *p* = 0.032), ADC_median_ (*r* = −0.422, adjusted *p* = 0.032), ADC_minimum_ (*r* = −0.390, adjusted *p* = 0.032), and ADC_rootmeansquared_ (*r* = −0.419, adjusted *p* = 0.032) (Figure 4). No significant correlations were found between ADC values, MVD, and CVF after FDR correction (adjusted *p* > 0.05) (Table 2).

### 3.4. Correlation Between T1 Values, Ki-67 Expression, MVD, and CVF

**Figure 5:**
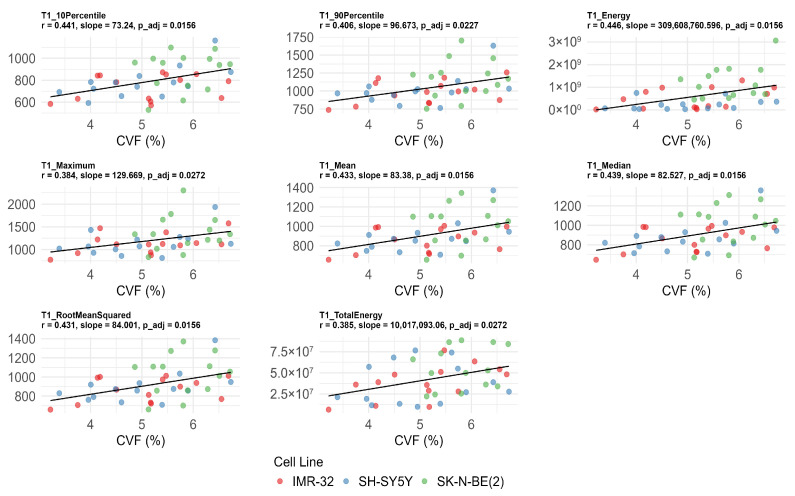
Significant correlations between native T1 histogram features and collagen volume fraction (CVF) after false discovery rate correction. The unit of T1_energy_ and T1_totalenergy_ is ms^2^ and ms^2^ × mm^3^, respectively, and the unit of remaining features is ms.

T1_10Percentile_ (*r* = 0.441, adjusted *p* = 0.016), T1_90Percentile_ (*r* = 0.406, adjusted *p* = 0.023), T1_energy_ (*r* = 0.446, adjusted *p* = 0.016), T1_maximum_ (*r* = 0.384, adjusted *p* = 0.027), T1_mean_ (*r* = 0.433, adjusted *p* =0.016), T1_median_ (*r* = 0.439, adjusted *p* = 0.016), T1_rootmeansquared_ (*r* = 0.431, adjusted *p* = 0.016), and T1_totalenergy_ (*r* = 0.385, adjusted *p* = 0.027) were also significantly positively correlated with CVF (Figure 5). No significant correlations were found between T1 values, Ki-67, and MVD after FDR correction (adjusted *p* > 0.05) (Table 3).

### 3.5. Inter-Observer Agreement

The ICCs for the two sets of ADC histogram measurements ranged from 0.83 to 0.99, with a mean of 0.93 (SD: 0.06), indicating good to excellent reliability. For the T1 histogram measurements, ICCs ranged from 0.85 to 1.00, with a mean of 0.96 (SD: 0.04), also indicating good to excellent reliability (Supplementary Table S1). Bland–Altman plots comparing the ADC and T1 histogram features between the two observers are shown in Supplementary Figures S2 and S3.

## 4. Discussion

### 4.1. Summary of Key Findings and Translational Implications

In this exploratory preclinical study, neuroblastoma xenograft models were established and subjected to DWI and native T1 mapping to generate maps of ADC and native T1 relaxation times. Histogram analyses were performed on these maps to extract quantitative histogram features, which were then correlated with Ki-67, MVD, and CVF. ADC histogram features showed significant associations with Ki-67 index, while T1 histogram features were significantly correlated with CVF after FDR correction. These findings suggest that MRI-based histogram analysis of ADC and native T1 mapping can noninvasively reflect tumor cell proliferation and key TME components in neuroblastoma. The complementary information derived from ADC and T1 mapping may contribute to noninvasive assessment of tumor aggressiveness, extracellular remodeling, and risk stratification, providing a methodological foundation for future diagnostic applications in clinical neuroblastoma imaging. Importantly, since the imaging protocol was implemented on a 3.0T clinical scanner, these techniques are readily translatable to patient studies.

### 4.2. Impact of MYCN Amplification on Tumor Biology

Neuroblastoma exhibits significant genetic and pathological heterogeneity. To investigate the impact of genetic background on tumor biology, we established xenograft models using both *MYCN*-amplified and non-amplified neuroblastoma cell lines. Our results indicate that differences in Ki-67 expression and MVD between the models were more significant than differences in CVF. Specifically, xenografts derived from *MYCN*-amplified cells exhibited higher Ki-67 indices and MVD compared to those derived from non-amplified cells. In our experiments, we found that SH-SY5Y subcutaneous xenografts were less tumorigenic and exhibited slower growth compared with SK-N-BE(2) and IMR-32. This biological heterogeneity may contribute to a wider distribution of proliferative activity, as reflected by the broader range of Ki-67 values. Additionally, SH-SY5Y is considered a less aggressive *MYCN*-non-amplified cell line, which might result in variable microenvironmental conditions within tumors and thus greater variability in proliferation rates. These findings are consistent with the established role of *MYCN* amplification in promoting tumor aggressiveness through increased cell proliferation and angiogenesis [33]. *MYCN* amplification, present in approximately 20% of neuroblastoma cases, is recognized as a key marker of poor prognosis and is strongly associated with aggressive disease behavior [34]. Collectively, our results highlight the distinct proliferative and TME characteristics associated with *MYCN* amplification in neuroblastoma.

### 4.3. ADC Histogram Features and Tumor Proliferation

Compared with single mean values measured within ROIs, histogram analysis provides more detailed and informative metrics. In the present study, Ki-67 expression was negatively correlated with multiple ADC histogram features, suggesting that higher tumor cell proliferation is associated with increased tissue cellularity, which restricts water diffusion and results in lower ADC values. These findings support the potential utility of ADC as a non-invasive imaging biomarker for evaluating tumor biological behavior. Consistently, multiple studies across various cancer types have reported negative correlations between ADC values and Ki-67 expression, with lower ADC values frequently observed in tumors exhibiting high Ki-67 levels [35,36,37]. As a marker of cell proliferation, elevated Ki-67 expression generally reflects increased tumor growth activity and potentially greater malignancy. Therefore, ADC values derived from DWI may serve as noninvasive indicators of tumor proliferative activity and aggressiveness in neuroblastoma. However, the strength of the correlation varies among different tumor types. A meta-analysis reported a pooled correlation coefficient between mean ADC and Ki-67 of ρ = −0.44 across all included tumors, with entity-specific coefficients ranging from −0.22 to −0.62 [38].

A previous study has shown that ADC histogram features differ between *MYCN*-amplified and non-amplified neuroblastomas [24]. Our results suggest that these differences in ADC histogram features may be attributable, at least in part, to variations in Ki-67 expression between *MYCN* status groups. However, only a subset of ADC features in our study showed a trend toward intergroup difference, none of which reached statistical significance. Given that ADC values can be influenced by the delineation of ROIs [39], variability in ROI segmentation methods may contribute to the observed differences between groups, highlighting a factor that warrants further investigation. Therefore, combined ADC and T1 histogram analysis may help distinguish neuroblastoma subtypes with different biological behaviors, particularly those associated with *MYCN* amplification. This could contribute to improved imaging-based risk assessment.

### 4.4. Variability in the Associations Between ADC and MVD

While ADC histogram features were significantly correlated with Ki-67, the relationship between ADC and MVD was weaker and inconsistent, with only ADC_skewness_ showing a significant association. Skewness can be used to measure the symmetry of the ADC value distribution. The observed negative correlation between ADC_skewness_ and MVD may indicate that increased vascular density contributes to a more symmetric distribution of water diffusion within the tumor. However, after FDR correction, no ADC features remained significantly associated with MVD. It is important to note that the relationship between ADC and MVD has varied across studies. For instance, Jung et al. [40] reported a significant correlation between mean ADC values and MVD in prostate cancer, suggesting a potential association between diffusion metrics and vascularity. Conversely, other studies have failed to identify such associations; for example, an investigation of whole-lesion ADC histogram features in head and neck squamous cell carcinoma found no statistically significant correlation between ADC values and MVD [41]. These discrepancies suggest that, while ADC provides a noninvasive tool for characterizing tumor biology, its utility as a surrogate marker for vascular density may be tumor-type specific and should be interpreted with caution. One possible explanation is that ADC, derived from the conventional mono-exponential model, is strongly influenced by tumor heterogeneity and is not well suited to capture microperfusion. Intravoxel incoherent motion (IVIM) imaging, which separates diffusion and perfusion components, may overcome this limitation. Indeed, IVIM parameters have been shown to correlate with MVD in rhabdomyosarcoma [31]. Future studies are therefore warranted to explore whether IVIM or other advanced diffusion models could provide more robust imaging biomarkers of vascularity in neuroblastoma.

### 4.5. T1 Histogram Features Reflecting Tumor Microenvironment

Our study revealed positive correlations between multiple histogram features derived from native T1 maps and both MVD and CVF, indicating that increased vascularization and collagen deposition are associated with higher native T1 values and related statistical metrics. These findings suggest that T1 histogram features may serve as noninvasive imaging biomarkers reflecting TME composition. Although direct associations between native T1 values and MVD have been rarely reported, tumor vascularity may influence T1 relaxation times by affecting tissue perfusion and interstitial fluid content. T1 relaxation time has been established as a reliable biomarker for assessing therapeutic response of TH-MYCN neuroblastoma in mice to the anti-angiogenic agent cediranib [42], implying a potential association between T1 relaxation and tumor vascular architecture and supporting the capacity of T1 mapping to sensitively capture treatment-induced vascular changes. In our study, a positive correlation was observed between T1_entropy_ and MVD. Entropy quantifies the complexity and heterogeneity of the T1 voxel intensity distribution. This finding suggests that tumors with higher MVD exhibit greater spatial heterogeneity in T1 relaxation times. The association may indicate that increased vascular complexity and perfusion variability contribute to the dispersion of T1 values within the TME. While several correlations between T1 map features and MVD did not retain statistical significance after FDR correction, the consistent trends observed across related parameters suggest underlying biological relevance rather than random variation. Given the exploratory and preclinical nature of this study, these results should be considered hypothesis-generating and warrant validation in larger datasets.

Additionally, we observed positive correlations between T1 histogram features and CVF even after FDR correction. These correlated histogram parameters were primarily those reflecting voxel-wise T1 intensity, such as T1_mean_ and T1_energy_, suggesting that native T1 values are influenced by collagen content within the TME. Mechanistically, the organization and density of collagen fibers can alter tissue water mobility and extracellular proton environments, thereby prolonging T1 relaxation times and increasing voxel-wise heterogeneity. The structural characteristics of the ECM, including collagen density and fiber organization, are closely correlated to tumor aggressiveness [43], as dense collagen can facilitate tumor initiation and progression by modifying mechanical properties and influencing cellular behavior [44]. Previous studies have demonstrated associations between native T1 values and tumor malignancy [45,46]. For example, in head and neck squamous cell carcinoma, the standard deviation of T1 measurements was significantly higher in malignant lesions compared to benign counterparts [47]. Similarly, in meningioma grading, several T1 histogram parameters, including T1_mean_, T1_maximum_, and T1_90Percentile_, were significantly elevated in high-grade tumors [48]. Collectively, these findings support the hypothesis that T1 histogram features derived from native T1 mapping may not only reflect ECM composition but also provide insights into tumor aggressiveness, potentially assisting in grading neuroblastoma and characterizing its microenvironment. Moreover, native T1 mapping is a non-contrast technique that avoids gadolinium administration, making it particularly suitable for pediatric patients, including those with renal impairment.

### 4.6. Limitations and Future Directions

Several limitations of this study should be acknowledged. First, subcutaneous xenograft models do not fully replicate the anatomical and microenvironmental context of neuroblastoma. While orthotopic models more closely resemble the clinical tumor microenvironment, they are technically challenging, costly, and less reproducible. In contrast, subcutaneous xenografts are relatively straightforward to establish, have a high success rate, and facilitate acquisition of high-quality imaging, which is particularly important for imaging-based studies [49]. Given the exploratory and preclinical nature of this work, we focused on subcutaneous xenografts derived from human neuroblastoma cell lines to investigate the potential imaging basis of native T1 mapping and to identify generalizable patterns. The heterogeneous origins of neuroblastoma, including the neck, mediastinum, abdomen, and pelvis, further emphasize the value of establishing broadly applicable principles. Second, this study relied solely on cross-sectional imaging without longitudinal assessment of treatment response or disease progression, which limits temporal insights. Third, external validation in patient cohorts and translation to clinical pediatric populations remain necessary. Finally, the use of five randomly selected fields for Ki-67 and Masson staining versus three hotspot regions for CD31 analysis may introduce potential sampling bias. Future studies employing automated digital pathology could provide a more unbiased and reproducible assessment of tumor histopathology.

## 5. Conclusions

In conclusion, this exploratory preclinical study demonstrates that ADC and native T1 mapping histogram analyses can noninvasively reveal distinct biological features of neuroblastoma xenografts. ADC histogram features were significantly associated with Ki-67 expression, while native T1 histogram features correlated with CVF after FDR correction. These findings provide preliminary evidence that combining ADC and T1 mapping may offer complementary insights into tumor proliferation and extracellular matrix composition. In addition, these noninvasive imaging biomarkers may have potential for characterizing *MYCN* amplification status and for exploring risk stratification in neuroblastoma. However, as a hypothesis-generating study in preclinical models, these results require further validation in larger patient cohorts.

## Figures and Tables

**Table 1:** Interpretations of histogram features.

Feature	Unit (ADC)	Unit (T1)	Interpretation
10Percentile	×10^−6^ mm^2^/s	ms	Intensity value at the 10th percentile of the voxel distribution
90Percentile	×10^−6^ mm^2^/s	ms	Intensity value at the 90th percentile of the voxel distribution
Energy	(×10^−6^ mm^2^/s)^2^	ms^2^	Sum of squared voxel intensities; higher values indicate greater overall intensity magnitude
Entropy	—	—	Measure of randomness or uncertainty in voxel intensities; higher values indicate greater heterogeneity
InterquartileRange	×10^−6^ mm^2^/s	ms	Difference between the 75th and 25th percentile intensity values
Kurtosis	—	—	Degree of peakedness of the intensity distribution; higher values indicate sharper peaks or more extreme values
Maximum	×10^−6^ mm^2^/s	ms	Maximum voxel intensity
MeanAbsoluteDeviation	×10^−6^ mm^2^/s	ms	Average absolute deviation of voxel intensities from the mean
Mean	×10^−6^ mm^2^/s	ms	Average voxel intensity
Median	×10^−6^ mm^2^/s	ms	Median voxel intensity
Minimum	×10^−6^ mm^2^/s	ms	Minimum voxel intensity
Range	×10^−6^ mm^2^/s	ms	Difference between maximum and minimum voxel intensities
RobustMeanAbsoluteDeviation	×10^−6^ mm^2^/s	ms	Average absolute deviation of voxel intensities from the mean
RootMeanSquared	×10^−6^ mm^2^/s	ms	Square root of the mean of squared voxel intensities; reflects overall magnitude
Skewness	—	—	Degree of asymmetry of the intensity distribution relative to the mean
TotalEnergy	(×10^−6^ mm^2^/s)^2^ × mm^3^	ms^2^ × mm^3^	Energy feature scaled by the total voxel volume
Uniformity	—	—	Sum of squared voxel intensities normalized by the total number of voxels; higher values indicate greater homogeneity
Variance	(×10^−6^ mm^2^/s)^2^	ms^2^	Mean of squared deviations of voxel intensities from the mean; reflects distribution spread

— indicates dimensionless.

**Table 2:** Correlations between ADC histogram features, Ki-67, MVD, and CVF.

ADC Features	Ki-67				MVD				CVF			
	*r*	Slope	*p*-Value	FDR *p*-Value	*r*	Slope	*p*-Value	FDR *p*-Value	*r*	Slope	*p*-Value	FDR *p*-Value
10Percentile	−0.397	−14.351	0.009	0.032 *	−0.188	−3.674	0.234	0.429	0.160	55.217	0.312	0.803
90Percentile	−0.394	−23.827	0.010	0.032 *	−0.186	−6.094	0.238	0.429	0.031	17.885	0.846	0.948
Energy	−0.095	−1,986,692.721	0.548	0.617	0.089	1,002,664.923	0.576	0.654	0.242	48,067,808.520	0.123	0.803
Entropy	−0.223	−0.015	0.156	0.234	−0.031	−0.001	0.846	0.875	0.020	0.013	0.898	0.948
InterquartileRange	−0.297	−5.599	0.057	0.110	−0.112	−1.150	0.478	0.615	−0.092	−16.635	0.561	0.803
Kurtosis	0.045	0.004	0.778	0.824	−0.270	−0.013	0.083	0.429	−0.021	−0.017	0.896	0.948
Maximum	−0.362	−27.082	0.019	0.048 *	−0.214	−8.692	0.173	0.429	−0.010	−7.395	0.948	0.948
MeanAbsoluteDeviation	−0.292	−3.191	0.061	0.110	−0.139	−0.822	0.381	0.571	−0.103	−10.738	0.517	0.803
Mean	−0.421	−18.547	0.005	0.032 *	−0.189	−4.509	0.230	0.429	0.111	46.511	0.485	0.803
Median	−0.422	−17.724	0.005	0.032 *	−0.186	−4.228	0.238	0.429	0.139	55.800	0.379	0.803
Minimum	−0.390	−13.191	0.011	0.032 *	−0.208	−3.811	0.186	0.429	0.145	46.933	0.359	0.803
Range	−0.251	−13.891	0.108	0.177	−0.163	−4.881	0.302	0.494	−0.103	−54.329	0.516	0.803
RobustMeanAbsoluteDeviation	−0.307	−2.463	0.048	0.108	−0.117	−0.508	0.461	0.615	−0.088	−6.740	0.580	0.803
RootMeanSquared	−0.419	−18.890	0.006	0.032 *	−0.193	−4.717	0.221	0.429	0.097	41.760	0.542	0.803
Skewness	−0.021	−0.001	0.894	0.894	−0.310	−0.009	0.046	0.429	−0.156	−0.081	0.324	0.803
TotalEnergy	−0.098	−930,886.009	0.539	0.617	0.088	452,553.132	0.581	0.654	0.240	21,859,364.951	0.126	0.803
Uniformity	0.215	0.001	0.172	0.238	0.025	0.000	0.875	0.875	−0.011	0.000	0.947	0.948
Variance	−0.183	−1916.782	0.246	0.316	−0.188	−1062.828	0.234	0.429	−0.220	−22,012.572	0.161	0.803

* indicates statistical difference after false discovery rate (FDR) correction.

**Table 3:** Correlations between T1 histogram features, Ki-67, MVD, and CVF.

T1 Features	Ki-67				MVD				CVF			
	*r*	Slope	*p*-Value	FDR *p*-Value	*r*	Slope	*p*-Value	FDR *p*-Value	*r*	Slope	*p*-Value	FDR *p*-Value
10Percentile	−0.248	−4.310	0.114	0.660	0.231	2.183	0.140	0.168	0.441	73.24	0.003	0.016 *
90Percentile	−0.124	−3.098	0.433	0.708	0.332	4.478	0.032	0.096	0.406	96.673	0.008	0.023 *
Energy	0.129	9,417,086.726	0.414	0.708	0.286	11,260,685.37	0.067	0.100	0.446	309,608,760.6	0.003	0.016 *
Entropy	0.127	0.007	0.422	0.708	0.368	0.011	0.016	0.075	0.256	0.14	0.102	0.154
InterquartileRange	0.096	0.662	0.546	0.754	0.265	0.993	0.090	0.115	0.116	7.687	0.463	0.490
Kurtosis	−0.219	−0.020	0.163	0.660	0.184	0.009	0.245	0.245	0.256	0.218	0.101	0.154
Maximum	−0.086	−3.058	0.586	0.754	0.359	6.884	0.019	0.075	0.384	129.669	0.012	0.027 *
MeanAbsoluteDeviation	0.075	0.290	0.639	0.766	0.318	0.669	0.040	0.100	0.168	6.23	0.287	0.345
Mean	−0.176	−3.556	0.264	0.660	0.288	3.14	0.065	0.100	0.433	83.38	0.004	0.016 *
Median	−0.174	−3.433	0.270	0.660	0.270	2.883	0.084	0.115	0.439	82.527	0.004	0.016 *
Minimum	−0.265	−4.038	0.090	0.660	0.220	1.817	0.161	0.182	0.332	48.339	0.032	0.063
Range	0.037	0.980	0.815	0.863	0.356	5.067	0.021	0.075	0.324	81.33	0.036	0.065
RobustMeanAbsoluteDeviation	0.109	0.308	0.493	0.739	0.286	0.44	0.066	0.100	0.117	3.156	0.462	0.490
RootMeanSquared	−0.172	−3.504	0.277	0.660	0.292	3.223	0.061	0.100	0.431	84.001	0.004	0.016 *
Skewness	−0.051	−0.003	0.750	0.844	0.184	0.005	0.244	0.245	0.064	0.031	0.689	0.689
TotalEnergy	0.166	452,989.793	0.293	0.660	0.436	643,801.091	0.004	0.071	0.385	10,017,093.06	0.012	0.027 *
Uniformity	−0.183	−0.001	0.246	0.660	−0.374	−0.001	0.015	0.075	−0.214	−0.007	0.173	0.239
Variance	0.019	26.533	0.904	0.904	0.290	216.393	0.062	0.100	0.192	2524.117	0.223	0.287

* indicates statistical difference after false discovery rate (FDR) correction.

## Data Availability

All data generated or analyzed during this study are included in this published article.

## Supplementary Materials

The following supporting information can be downloaded at: https://www.mdpi.com/article/10.3390/cancers17213433/s1, Figure S1: Comparisons of collagen volume fraction (CVF), Ki-67 expression, and microvessel density (MVD) among IMR-32, SH-SY5Y, and SK-N-BE(2) groups; Figure S2: Bland–Altman plots of apparent diffusion coefficient (ADC) histogram features between the two observers. The unit of energy and variance is (×10^−6^ mm^2^/s)^2^, and the unit of totalenergy is (×10^−6^ mm^2^/s)^2^×mm^3^. The unit of entropy, kurtosis, skewness, and uniformity is dimensionless. The unit of remaining features is ×10^−6^ mm^2^/s; Figure S3: Bland–Altman plots of native T1 histogram features between the two observers. The unit of energy and variance is ms^2^, and the unit of totalenergy is ms^2^×mm^3^. The unit of entropy, kurtosis, skewness, and uniformity is dimensionless. The unit of remaining features is ms; Table S1: Intra-class correlation coefficients of ADC and T1 features.

## Abbreviations

The following abbreviations are used in this manuscript:

TME	tumor microenvironment
ECM	extracellular matrix
MVD	microvessel density
MRI	magnetic resonance imaging
ROI	region of interest
T1WI	T1-weighted imaging
T2WI	T2-weighted imaging
DWI	diffusion-weighted imaging
ADC	apparent diffusion coefficient
TR	repetition time
TE	echo time
FOV	field of view
VFA	variable flip angle
CVF	collagen volume fraction
ANOVA	one-way analysis of variance
SD	standard deviation
ICCs	intra-class correlation coefficients
IVIM	intravoxel incoherent motion
FDR	false discovery rate
SPGR	spoiled gradient-recalled echo
