# Supplementary material for: Apparent Diffusion Coefficient and Native T1 Mapping Histogram Analyses Reveal Tumor Proliferation and Microenvironment in Neuroblastoma Xenografts
Source: Cancers (Basel). 2025 Oct 26;17(21):3433. doi: 10.3390/cancers17213433 (PMC12606753; doi:10.3390/cancers17213433)
Supplement: Supplementary file 1 [file cancers-17-03433-s001.zip › cancers-3860587-supplementary.pdf]

Supplementary Materials

Supplementary Table S1. Intra-class correlation coefficients of ADC and T1 features.

**Histogram features**	**ADC-based ICC (95% CI)**	**T1-based ICC (95% CI)**
10Percentile	0.993 (0.988-0.996)	0.988 (0.978-0.994)
90Percentile	0.905 (0.831-0.948)	0.998 (0.997-0.999)
Energy	0.855 (0.746-0.919)	0.938 (0.889-0.966)
Entropy	0.839 (0.720-0.910)	0.969 (0.943-0.983)
InterquartileRange	0.972 (0.948-0.985)	0.977 (0.957-0.987)
Kurtosis	0.834 (0.712-0.907)	0.928 (0.871-0.961)
Maximum	0.992 (0.986-0.996)	0.994 (0.990-0.997)
MeanAbsoluteDeviation	0.985 (0.972-0.992)	0.981 (0.965-0.990)
Mean	0.981 (0.965-0.990)	0.997 (0.995-0.999)
Median	0.991 (0.983-0.995)	0.997 (0.995-0.999)
Minimum	0.983 (0.968-0.991)	0.877 (0.783-0.932)
Range	0.978 (0.959-0.988)	0.956 (0.920-0.976)
RobustMeanAbsoluteDeviation	0.967 (0.939-0.982)	0.979 (0.962-0.989)
RootMeanSquared	0.973 (0.950-0.985)	0.998 (0.996-0.999)
Skewness	0.918 (0.853-0.955)	0.851 (0.741-0.917)
TotalEnergy	0.858 (0.750-0.921)	0.937 (0.885-0.966)
Uniformity	0.925 (0.865-0.959)	0.957 (0.921-0.977)
Variance	0.885 (0.797-0.936)	0.982 (0.966-0.990)

ADC, apparent diffusion coefficient; CI, confidence interval; ICC, intra-class correlation coefficient

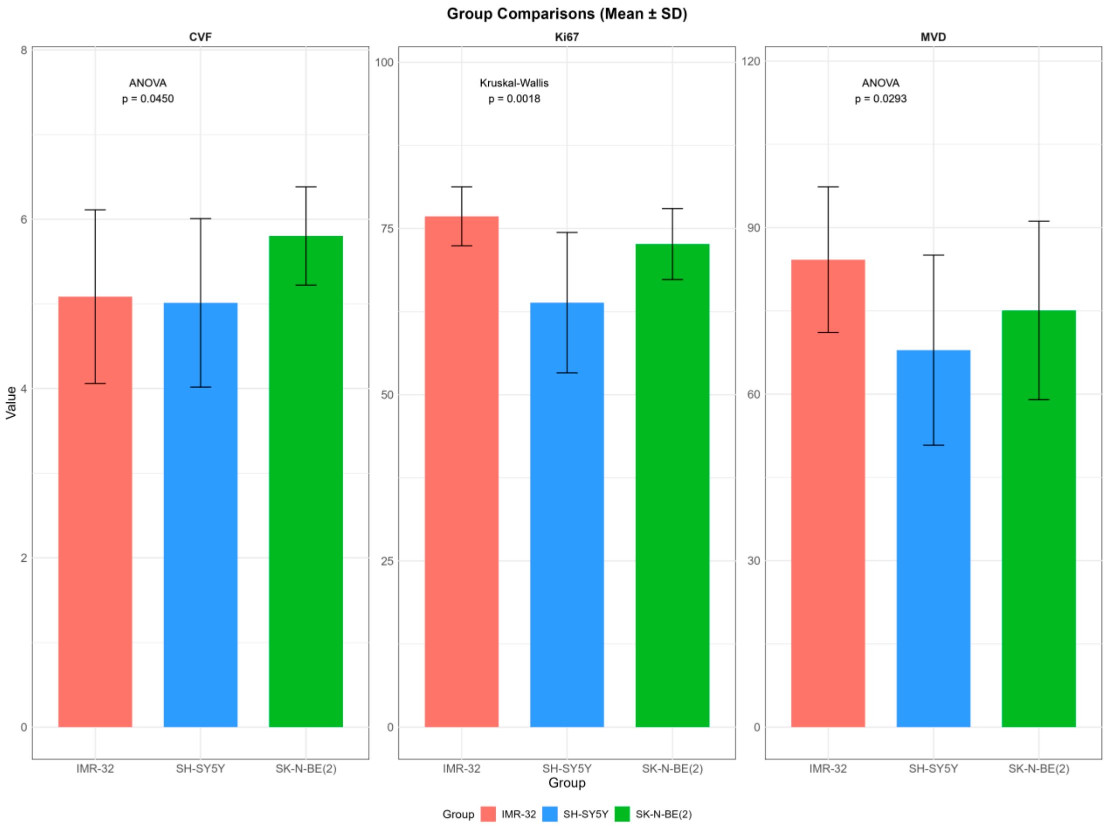

Supplementary Figure S1. Comparisons of collagen volume fraction (CVF), Ki-67 expression, and microvessel density (MVD) among IMR-32, SH-SY5Y, and SK-N-BE(2) groups.

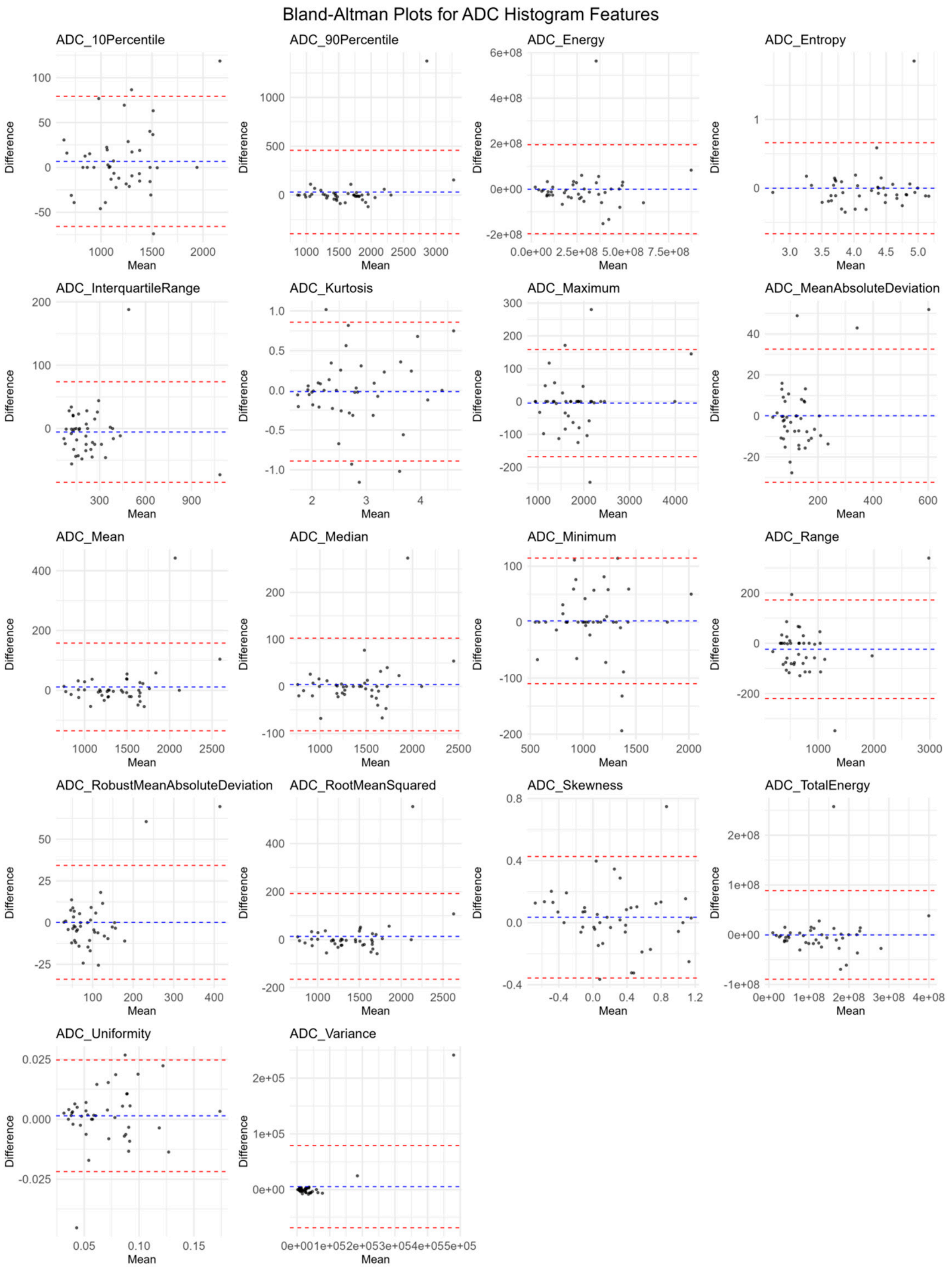

Supplementary Figure S2. Bland-Altman plots of apparent diffusion coefficient (ADC) histogram features between the two observers. The unit of energy and variance is ($\times10^{-6}$ $mm^2/s)^2$, and the unit of totalenergy is ($\times10^{-6}$ $mm^2/s)^2\times mm^3$. The unit of entropy, kurtosis, skewness, and uniformity is dimensionless. The unit of remaining features is $\times10^{-6}$ mm^2/s.

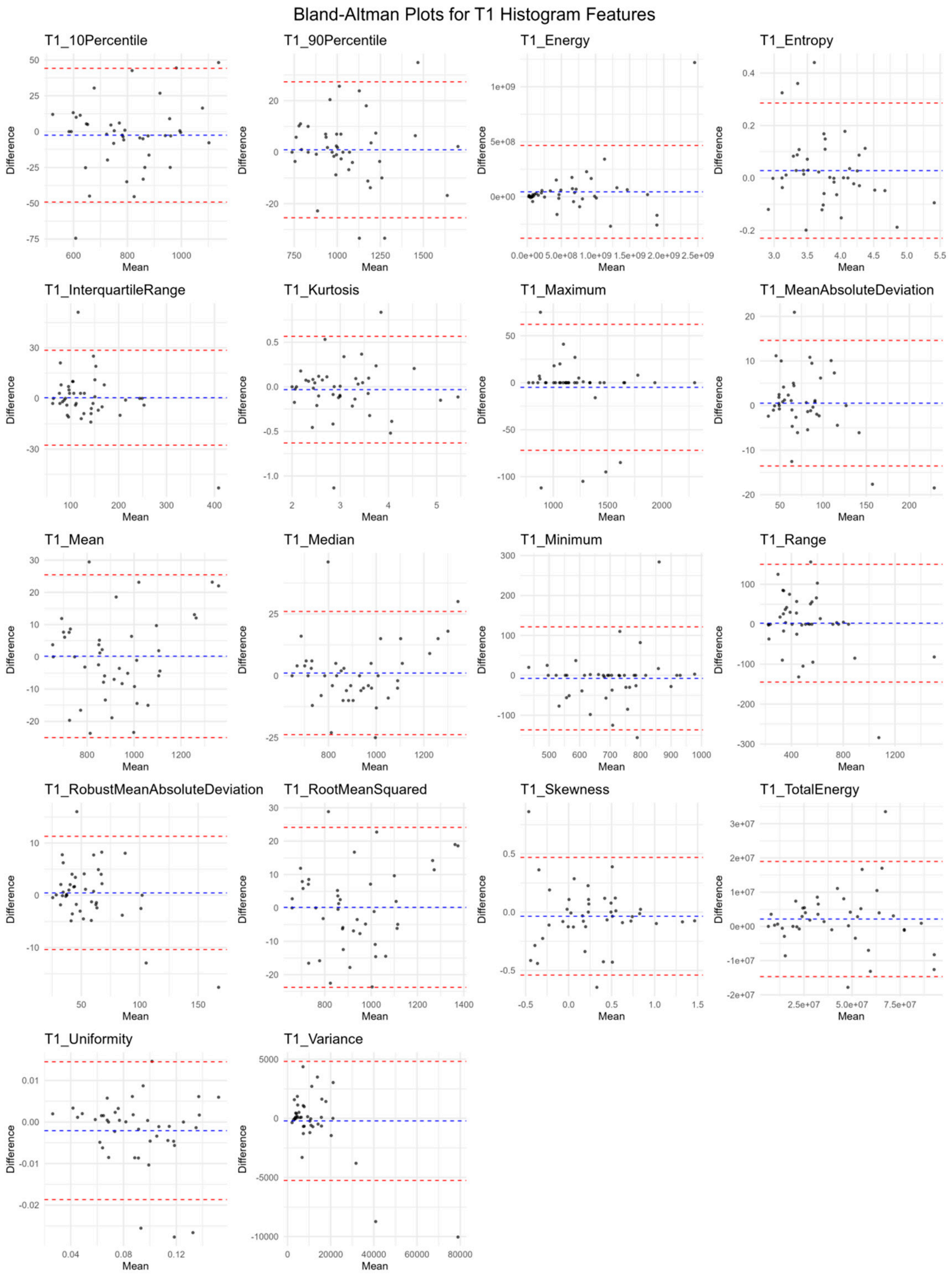

Supplementary Figure S3. Bland-Altman plots of native T1 histogram features between the two observers. The unit of energy and variance is ms^2, and the unit of totalenergy is $ms^2 \times mm^3$. The unit of entropy, kurtosis, skewness, and uniformity is dimensionless. The unit of remaining features is ms.
